# Both the domain-general and the mentalising processes affect visual perspective taking

**DOI:** 10.1177/17470218221094310

**Published:** 2022-06-02

**Authors:** Gabriele Pesimena, Alessandro Soranzo

**Affiliations:** 1 Centre for Behavioural Science and Applied Psychology, Sheffield Hallam University, Sheffield, UK; 2 School of Psychological Sciences, University of Bristol, Bristol, UK

**Keywords:** Visual perspective taking, attention, dot-perspective task, spatial cueing, mentalising, Bayesian statistics

## Abstract

People’s attention cannot help being affected by what others are looking at. The dot-perspective task has been often employed to investigate this visual attentional shift. In this task, participants are presented with virtual scenes with a cue facing some targets and must judge how many targets are visible from their own or the cue perspective. Typically, this task shows an interference pattern: Participants record slower reaction times (RTs) and more errors when the cue is facing away from the targets. Interestingly, this occurs also when participants take their own perspective. Two accounts contend the explanation of this interference. The mentalising account focuses on the social relevance of the cue, while the domain-general account focuses on the directional features of the cue. To investigate the relative contribution of the two accounts, we developed a Social_Only cue, a cue having only social features and compared its effects with a Social+Directional cue, which had both social and directional features. Results show that while the Social+Directional cue generates the typical interference pattern, the Social_Only cue does not generate interference in the RTs, only in the error rate. We advance an integration between the mentalising and the domain-general accounts. We suggest that the dot-perspective task requires two processes: an orienting process, elicited by the directional features of the cue and measured by the RTs, and a decisional process elicited by the social features of the cue and measured also by the error rate.

## Introduction

*“That’s how I do this life sometimes by making the ordinary just like magic and just like a card trick and just like a mirror and just like the disappearing. Every Indian learns how to be a magician and learns how to misdirect attention.”*

The Lone Ranger and Tonto Fistfight in Heaven

Attention allows individuals to efficiently process information by allocating cognitive resources to specific information, stimuli, or location (Pesimena et al., 2019). For centuries, magicians have used different techniques to direct our attention and control what we can and cannot see. Like the magicians and their tricks visual or auditory cues can direct attention towards certain stimuli. These cues may produce a reflexive, rather than voluntary, attentional shift. Although a voluntary shift of attention depends on our expectations and intentions, a reflexive attentional shift is generated by unforeseen changes in the environment, such as an abrupt onset of a stimulus, or by directional cues capable of shifting attention towards where they are pointing. An interesting case is when the directional cue has social relevance. In this case, some authors interpret the attentional shift as the result of visual perspective taking (hence the title of this article). Other authors, however, interpret this shift as the result of general-domain processes. This article assesses the two interpretations.

To experimentally investigate this phenomenon, Samson et al. (2010) devised an ad hoc dot-perspective task consisting of a three-dimensional virtual room with the back, left, and right walls visible on the computer screen. In the centre of the room, a human-shaped avatar serves as a directional cue the purpose of which is to direct attention towards either the left or the right wall, depending on which side it is facing. During the experiment, a number of discs appear on the left, on the right, or both the walls. Before the room and the avatar are shown, two prompts are presented to the participant (1) the prompts YOU or SHE, which instruct the participants to take either their own or the avatar’s perspective, respectively, and (2) a number indicating how many discs may be presented. The participant’s task is to respond as quickly as possible via a keypress whether the number of discs visible from the instructed perspective is the same as the prompted one.

**Figure 1.**
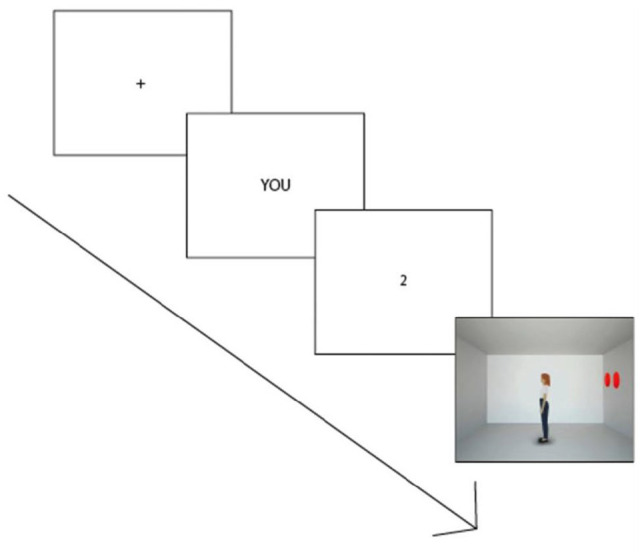
The timeline of the dot-perspective task as ideated by Samson et al. (2010) after the presentation of a fixation point, participants are instructed to take a perspective (either YOU or SHE), then a number between 0 and 3 appears and finally the room with the avatar facing one of the walls appears. Participants are requested to press a key on the keyboard for a YES (meaning that the number of discs visible from the prompted perspective is correct) or another key for a NO (meaning that the number of discs visible from the prompted perspective is incorrect). In this example, the correct answer would be YES because although the avatar sees an empty wall, the participant (prompted with YOU in this case) sees two discs as prompted.

**Figure 2.**
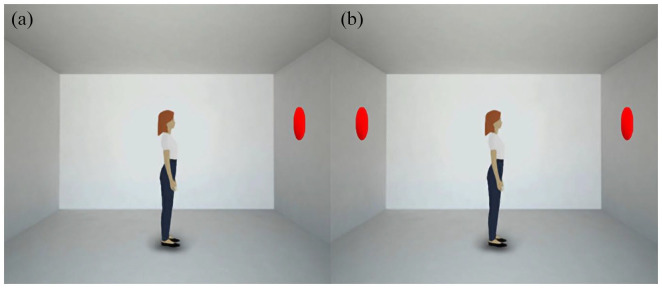
Types of trials in the dot-perspective task (a) Example of a Consistent trial: both the participant and the avatar see the same number of discs (one in the figure). (b) Example of an Inconsistent trial: the participant sees two discs while the avatar sees only one disc.

Figure 1 shows the timeline of the dot-perspective task when the prompted perspective is Self (induced by the prompt YOU) and the number of prompted discs is 2. In this case, the correct answer is YES because the number of discs visible from the participant’s viewpoint is the same as the prompted number. The correct answer would have been NO if the prompted perspective was the avatar’s one (that would have been induced by the prompt SHE). Indeed, in Figure 1, the avatar is facing an empty wall, with no visible discs from its viewpoint. While the participant can always see the total number of discs, the avatar cannot, thus generating Consistent trials—the avatar and the participant see the same numbers (Figure 2a).—and Inconsistent trials—the avatar sees a reduced number of discs (Figure 2b). Reaction times (RTs) and error rates are the dependent variables measured by the task.

Using this paradigm, an interference pattern emerges in inconsistent trials. Participants usually exhibit longer RTs and more errors than in consistent trials. This interference occurs both when participants take the avatar perspective and, interestingly, also when they take their own perspective.

The interference occurring when taking the other perspective is unanimously interpreted considering the Theory of Mind (the ability to infer somebody else mental state; Premack & Woodruff, 1978) and is known as an egocentric intrusion (from the Latin *ego* “I”). As anticipated, there is no consensus on the cause of the interference occurring when participants report what they see themselves. On the one side, the mentalising account explains this interference suggesting that when judging their own perspective, the participants reflexively take into consideration the perspective of the avatar (the Other). In other words, due to the social nature of perception and action, participants cannot prevent themselves from mentalising what the others are thought to see, i.e., a visual perspective-taking process (e.g., Capozzi et al., 2014; Furlanetto et al., 2016; Morgan et al., 2018; Nielsen et al., 2015). Building upon the notion of egocentric intrusion and the Theory of Mind, the mentalising account named this phenomenon *altercentric* intrusion (from the Latin alter “Other”).

On the other side, the domain-general account suggests that the other’s directional features such as their posture and face orientation are the cause of this interference (e.g., Cole et al., 2015; Cole & Millett, 2019; Heyes, 2014; Langton, 2018; Pesimena et al., 2019) disputing the involvement of Theory of Mind and the concept of altercentric intrusion.

The mentalising account is supported by the evidence that if the human avatar (the Other) is replaced by a rectangle distractor (as in Samson et al., 2010) the interference disappears, indicating that the social relevance of the cue is necessary for the interference to occur. The domain-general account instead is supported by the evidence that the interference can be generated by directional cues that do not possess a mental state such as arrows (Santiesteban et al., 2015), cameras (Wilson et al., 2017), or even chairs (MacDorman et al., 2013). Oppositely to previous evidence, this shows that the social relevance of the cue is not necessary to generate interference.

A digression is necessary here, while the interference occurring when participants take their own perspective emerges both in RTs and errors in most of the studies, discordant results between the two measures emerged at times. For example, O’Grady et al. (2020), Langton (2018), and Cole et al. (2016) found interference in the RTs but not in the errors. Authors did not pay too much attention to this discordance and interpreted their results ignoring the error rate. This issue will be further discussed later in the article.

The debate is still ongoing as to which of these processes are at play. To test the two accounts, Michael and D’Ausilio (2015) suggested manipulating participants’ beliefs about the avatar being able to see. This should modulate the interference pattern. This suggestion has been received by different authors, but the results were far from conclusive in favour of either accounts. While an avatar believed to be unable of seeing still generated interference in Cole et al. (2015) and Wilson et al. (2017), it did not in Furlanetto et al. (2016). It seems therefore that both the manipulation of the participants’ beliefs and the use of cues without social features have been inconclusive. Hence, neither of the two accounts was able to fully rule out the other. In light of this, Capozzi and Ristic (2020) suggested an integrated approach: both domain-general and mentalising processes may play a role in the reflexive attentional shift. While directional cues may generate interference, a mental state attribution would modulate its magnitude.

**Figure 3.**
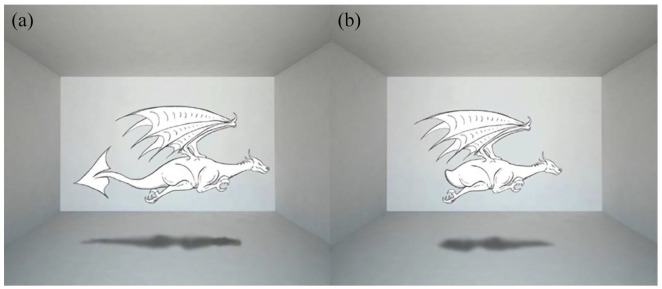
Cues used in this study (a) Social_Only cue: A dragon with an arrowed shaped tail pointing in the opposite direction of the muzzle. The role of the tail is to contrast the directional features of the dragon’s muzzle leaving only its social features. (b) Social+Directional cue: same dragon but without the arrowed shaped tail. The directional feature of the muzzle is not contrasted by any directional features.

In this study, we test the role of the mentalising and of the domain-general processes in generating attentional interference and their relative contribution. To do this, we focus on the features of the cue. In previous cues, the directional and social features that elicited domain-general and mentalising processes were conjugated. That is, consider the avatar of Figure 2, the directional features—signified by its posture—and the social features—signified by its viewpoint—both indicate the same direction. As it is difficult, if not impossible, to disentangle the social from the directional feature of the avatar, we reason that it can be possible to cancel out or attenuate the directional feature by providing the avatar with contrasting directional information. To this end, we developed a bidirectional cue. This cue consists of a dragon with an arrow-shaped tail pointing in the opposite direction of its muzzle (Figure 3a). In the dragon with the arrow-shaped tail, the social features of the muzzle (viewpoint) are isolated because the conjugated directional features are contrasted by the directional features of the tail.^1^ The purpose of the tail was to cancel or attenuate the directional features of the dragon’s posture (i.e., muzzle, wings, paws, etc.). For this reason, the size of the tail was chosen to achieve similar directional effects to those of the posture. This was assessed by means of a preliminary experiment using the Posner spatial cueing paradigm (Posner & Cohen, 1984). In this task, participants are presented with a directional cue followed by a target stimulus which can appear either in the cued location (congruent) or in the opposite (incongruent). Participants are asked to detect as quickly as possible when the target appears. Typically, this task shows a cueing effect: slower RTs in the incongruent condition. No cueing effect emerged in this task when the dragon with an arrow-shaped tail was employed as a cue, while the effect emerged when the tail was removed. Thus, confirming the role of the tail to cancel out the directional features of the posture (see online Supplementary Material A).

As the directional features of this cue are cancelled out or attenuated by the tail, this cue was referred to as the *Social_Only* cue.

The reason for choosing a dragon with an arrow-shaped tail instead of any other bidirectional cue or combinations of cues (e.g., a human-avatar and an arrow pointing in the opposite direction) is because the dragon has the following desiderata:

- Fantasy creatures, such as a dragon, can orient attention in the same way as human avatars (MacDorman et al., 2013).
- As the dragon is present and inherited in every culture (Blust, 2000; Khalifa-Gueta, 2018), attention orientation is not affected by the lack of familiarity with the cue.
- As the arrow-shaped tail follows the body harmoniously, it is not recognised as an additional cue and attention orientation is not affected by the complexity of a scene with multiple cues.

We compare the effects of the *Social_Only* cue with those of a similar dragon without the arrow-shaped tail (Figure 3b).^2^ In this case, the directional features of the body’s posture are not contrasted by any other directional features. Therefore, both social and directional features of the head conjugately orient attention. We refer to this cue as the *Social+Directional* cue. The preliminary experiment confirmed that this cue directs attention (see online Supplementary Material A).

### Account’s predictions

Hence, by using the aforementioned cues in the dot-perceptive task, it is possible to clarify the relative contribution of social and directional features and discriminate the predictive validity of the mentalising and domain-general accounts in generating attentional interference. Specifically, when participants are judging their own perspective, the two accounts make different predictions:

- The mentalising account predicts that both *Social_Only* and *Social+Directional* cues generate the same amount of interference. This is because according to this account, the directional features on their own are not sufficient to generate interference, but the social features need also to be present in the cue.
- The domain-general account predicts that the *Social_Only* cue should generate less or no interference because the directional features of the body posture are cancelled out or attenuated by the tail, leaving no directional features to orient attention.^3^

So far, it was assumed that an interference emerges in both the RTs and error measures. However, this might not be the case. As mentioned, discordant results between RTs and errors emerged in the studies of Cole et al. (2015), Langton (2018), and O’Grady et al. (2020), where an interference emerged in the RTs but not in the error rate. In this regard, Prinzmetal et al. (2005) suggest that there are two processes whereby spatial cues capture attention: a voluntary process, affecting both RTs and errors, and an involuntary process, affecting RTs only. In agreement with Prinzmetal et al.’s suggestion, it can be hypothesised that the involuntary process, affecting RTs only, is driven by the directional features of the cue, while the voluntary process, affecting both RTs and errors, is driven by the social features of the cue. If this was the case, with the *Social_Only* cue, the interference should emerge in the error rate, while it should be reduced in the RTs because only the voluntary process is at play. This result would support the integrated approach advanced by Capozzi and Ristic (2020) because it would imply that both the mentalising and the domain-general processes are playing a role in the dot-perspective task.

To assess these predictions, we adopted the Bayesian rather than the frequentist approach. The Bayesian approach can obtain evidence for a null result and discriminate between the absence of evidence and evidence of absence (Dienes, 2014). In addition, the Bayesian approach provides a credible interval indicating the points of the distribution of the variable under consideration that are most credible. This allows a weighted evaluation of the results rather than a dichotomous decision. These characteristics are appealing for the aim of assessing the mentalising and the domain-general accounts because (1) both accounts draw conclusions based on a null effect and (2) it allows an estimation of their relative contribution.

### Ethics

This project was approved by the Psychology Research Ethics Panel at Sheffield Hallam University (nr. ER12646660).

## Methods

### Sampling plan and stopping rule

The Sequential Bayes Factors (SBF) procedure was followed to define the sample size (Schönbrodt et al., 2017). The SBF involves the calculation of subsequent Bayesian Factors (BF) after the collection of each new data, up to the achievement of a BF value determined a priori. Jeffreys (1961) suggests continuing the data collection until a BF of 10 in favour of one or the other hypothesis is reached. This value is considered “strong” evidence in favour of the considered hypothesis. Before starting the experiment, we had planned to suspend data collection based on the following “stopping rules”:

1. Achievement of a minimum number of 16 participants per each type of cue (i.e., the same number of participants employed by previous research on attentional interference, [e.g., Samson et al., 2010)]. Moreover, this figure is supported by a prospective power analysis conducted by Wilson et al. (2017) which also indicated that the sample size of 16 participants per condition would provide strong power [.8] to detect the expected effect.
2. Achievement of a BF in favour of one of the hypotheses for either RTs or error rate equal to 10 (as suggested by Jeffreys, 1961). Thus, we continued data collection until we reached our predetermined stopping criterion at the point of checking. Sampling was stopped after collecting 16 participants per each type of cue as one of the BF_10_ was higher than 10 (specifically, the BF_10_ of the interference for the *Social+Directional* cue was equal to 141).

### Participants

Thirty-two participants took part in this study (age range 22–47) of which 20 were females. Participants were naïve to the purpose of the study and received no remuneration for taking part. Informed consent was obtained from each participant through the Qualtrics online platform (https://www.qualtrics.com) in accordance with the University’s ethical procedures.

### Design

The variables used in the study were: Consistency (inconsistent vs. consistent), Perspective (self vs. other) and Types of cue (*Social_Only* vs. *Social+Directional*). While the variables Consistency and Perspective were measured within-subjects—as the dot-perspective task requires—the variable Types of cue was measured between-subjects. This was to control for the “experimental subordination” phenomenon (Asch, 1956; Gilchrist, 2020). If the same participants would have seen a dragon with and without the tail, they might have adjusted their answers according to what they thought they were expected to respond.

### Stimuli and procedure

Stimuli created using Adobe Photoshop (version: 21.1.2) were presented using Psychopy (version: 3) software and its online repository Pavlovia (Peirce et al., 2019). Due to the current COVID-19 situation, the use of Pavlovia via browser was the best option to carry out the study. As shown in Bridges et al. (2020), PsychoPy/PsychoJS recorded a precision of under 4 ms in every browser/OS combination; the precision improved even more (less than a millisecond) when Chrome is used as a browser in either Windows or Linux. Participants were therefore instructed on the information page to run the experiment using these OS and browser; furthermore, they were instructed not to run any other software or browser pages while running the experiment as these may have interfered and caused lags in recording response times. Stimulus presentation followed the dot-perspective task standard sequence (e.g., Samson et al., 2010). At the beginning of each trial, participants were presented with a fixation cross for 750 ms. After 500 ms the pronouns YOU or DRAGON appeared on-screen and were visible for 750 ms. Participants were instructed so that with the prompt YOU they should adopt their own perspective (self), while with the prompt DRAGON, they should adopt the cue perspective (other).^4^ Following the prompt and another gap of 500 ms, a number, either 1, 2, or 3, was presented for 750 ms. This number indicated the discs that participants were asked to verify if visible from the prompted perspective. The cue was then presented at the centre of the screen until the participant responded by pressing on the keyboard either A (YES; the stated number of discs is visible from the given perspective) or L (NO; the stated number of discs is not visible from the given perspective). If the participant did not respond within 2,000 ms, the next trial started, and the trial was considered an error. The combination of types of trials (consistent vs. inconsistent) and perspective (self vs. other) options generated four different types of trials per each types of cue. Furthermore, trials can be divided into YES and NO responses. While all consistent YES, inconsistent YES, and inconsistent NO trials require the participant to evaluate at least one perspective a potential confound arises from all consistent NO trials and inconsistent NO trials as the number presented to the participant did not match the number of discs visible from either perspective. For this reason, only the YES trials were included in the analysis (see Samson et al., 2010). In total, 80 trials were presented to each participant. These comprised 36 YES and 44 NO response trials. Thirty-six were consistent trials, 36 were inconsistent trials, and 8 were fillers, in which no discs were presented. Furthermore, 40 trials had as prompted perspective YOU, while the remaining 40 had DRAGON. Before the start of the experiment, participants took part in a small practice of 12 trials to familiarise themselves with the task. The experiment lasted on average 15 min.

### Data availability statement

Dataset and code for analysis are provided as part of the replication package together with an Rmarkdown version of this article are available at https://osf.io/62kd4/.

## Results

### Descriptive statistic

**Figure 4.**
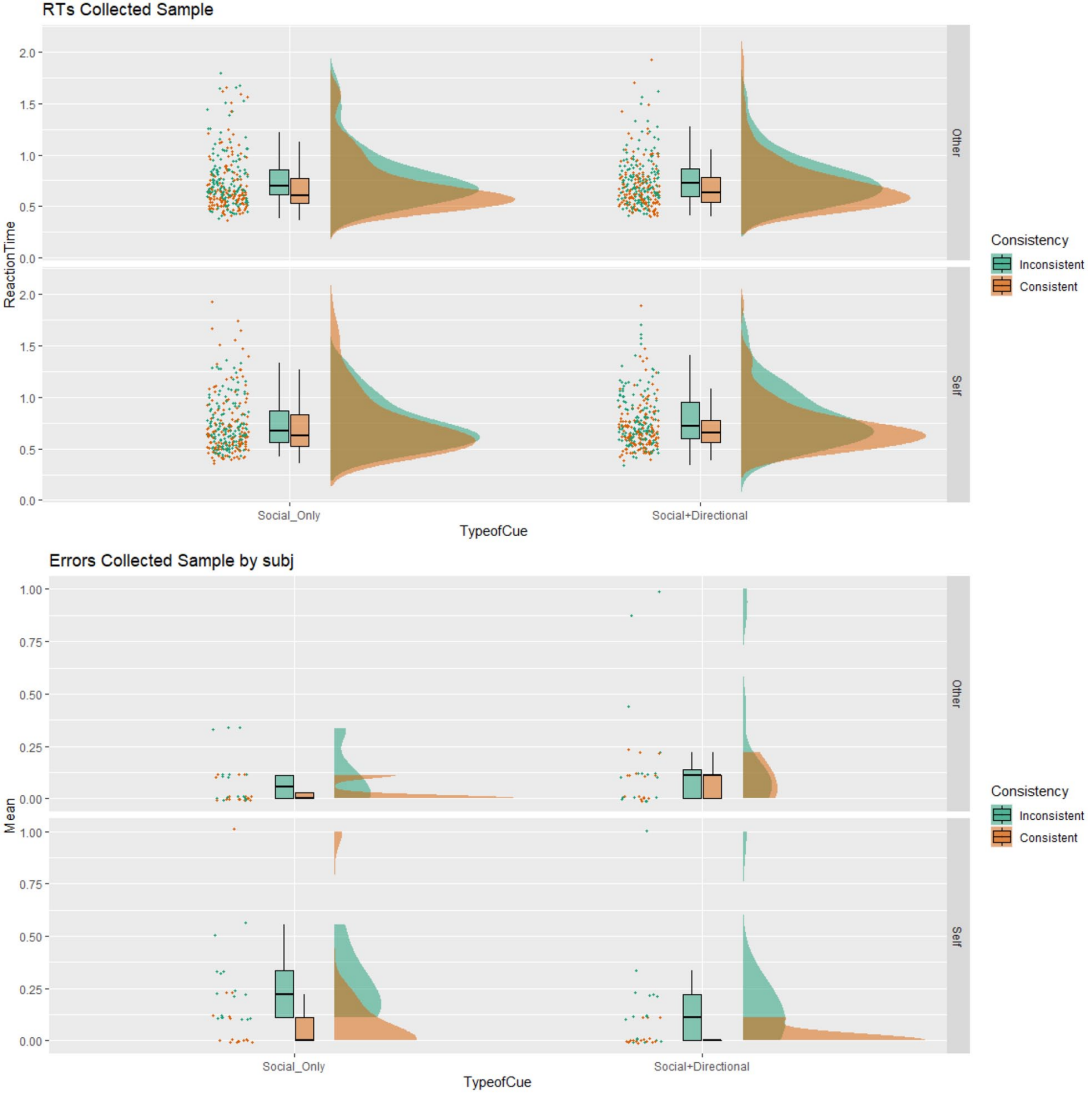
Rain plots reporting Mean and *SE* of distribution for sample’s RTs on the left and error rate on the right for each combination of stimulus presentation (consistent vs. inconsistent) and perspective adopted (self vs. other) for the two types of cue (Social+Directional vs. Social_Only). Error rates are averaged also by Subject.

Means and standard deviations for both RTs and error rates are shown in Table 1 and Figure 4. As per Whelan (2008), trials in which RTs are faster than 100 ms should be considered non-genuine. No RTs lower than 100 ms were present in this study. No trimming was conducted on higher reaction times, given the imposed cut-off of 2,000 ms on all trials.

**Table 1.** Mean and *SD* for RTs and error rate.

RTS				
Perspective	Consistency	Type of cue	Mean (s)	*SD*
Other	Inconsistent	*Social_Only*	0.780	0.276
		*Social+Directional*	0.761	0.231
	Consistent	*Social_Only*	0.695	0.262
		*Social+Directional*	0.695	0.242
Self	Inconsistent	*Social_Only*	0.731	0.229
		*Social+Directional*	0.789	0.259
	Consistent	*Social_Only*	0.728	0.306
		*Social+Directional*	0.708	0.231
Error rate				
Perspective	Consistency	Type of cue	Mean (s)	*SD*
Other	Inconsistent	*Social_Only*	0.097	0.297
		*Social+Directional*	0.196	0.398
	Consistent	*Social_Only*	0.028	0.165
		*Social+Directional*	0.084	0.278
Self	Inconsistent	*Social_Only*	0.232	0.424
		*Social+Directional*	0.167	0.374
	Consistent	*Social_Only*	0.105	0.307
		*Social+Directional*	0.021	0.144

RTs: reaction times.

As it can be seen, for the RTs an interference pattern (intended as the mean difference between the inconsistent and the consistent trials) emerged for both the level of the Perspective variable. In addition, for the self, the interference was much higher in the *Social+Directional* cue than in the *Social_Only* cue, where it was negligible (0.081 and 0.003 s on average, respectively).

A similar interference pattern emerged for the error rate. However, for the Self condition of the Perspective variable, the interference was alike for the two types of cue, with a mean error rate of 0.146 (*SD* 0.23) and 0.127 (*SD* 0.12) for the *Social+Directional* and *Social_Only* cues, respectively.

### Data analysis

To enable generalisation across stimuli and participants, data were analysed with mix-models (Judd et al., 2012); specifically, Bayesian mix-models were created in the Stan computational framework (Carpenter et al., 2017) accessed with the high-level interface “brms” package 2.10.0 (Bürkner, 2017, 2018) in R version 3.6.2 (R Core Team, 2020). Two models were run, one for the RTs and another for the error rate. For both models, the variables Perspective, Consistency, and Types of cue—together with their interaction—were inputted as population-level factors and the variable Subject as a group-level factor. Moreover, as each combination of the conditions was presented in more than one trial, the variable Trials was also inputted in the models as a group-level factor nested within the variable Subject. The two models were therefore similar in their formulae; however, we utilised the Weibull family distribution for the RTs (Logan, 1992; Palmer et al., 2011; Rouder et al., 2005) and the Bernoulli family distribution for the error rate (Bürkner, 2018). For testing opposite predictions, we set flat priors for the population-level effects and weakly informative priors for the intercept [student_t(3, 0.7, 2.5)] and for the group-level effects [student_t(3, 0, 2.5)]. For model estimation, four chains with 4,000 iterations (2,500 warmup) were used. Convergence was checked via Gelman and Rubin (1992) convergence statistics (Rhat close or equal to 1.0) and by visual inspection of the posterior distribution of all the coefficients and their chain convergence.

### Reaction time analysis

**Figure 5.**
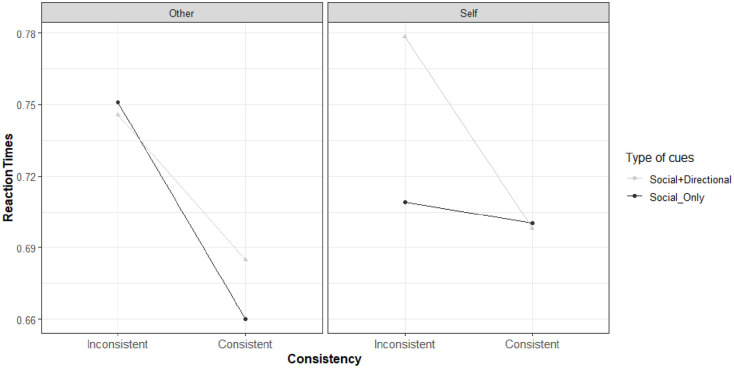
Estimated marginal means for each combination of stimulus presentation (inconsistent vs. consistent) and Types of cue (Social+Directional vs. Social_Only) for perspective adopted (self vs. other).

Table 2 shows the results of the Bayesian mixed-effects model. Figure 5 shows the estimated marginal means of the interaction between Consistency and Perspective split by the two types of cue. A main effect of Perspective emerged, with shorter RTs for the Self trials (−0.06, *SE* 0.03, 95% CI [−0.11, −0.01]). A main effect of Consistency also emerged, with shorter RTs for the Consistent trials (−0.13, *SE* 0.03, 95% CI [−0.18, −0.08]). Furthermore, an effect of the interaction between Perspective and Consistency emerged, with longer RTs in the Self-Consistent trials (0.12, *SE* 0.04, 95% CI [0.04, 0.19]). Finally, an effect of the interaction between Perspective and Types of cue emerged, with longer RTs in the Self-*Social* *+* *Directional* trials (0.10, *SE* 0.04, 95% CI [0.03, 0.18]). There was also an effect of the three-way interaction that is further explored in the planned comparisons.

**Table 2.** Population-level effects of the brms model.

Covariate	Estimate	Est. error	l-95% CI	u-95% CI
Intercept	−0.29	0.07	−0.41	−0.16
PerspectiveSelf	−0.06	0.03	−0.11	−0.01
ConsistencyConsistent	−0.13	0.03	−0.18	−0.08
TypesofCueSocialPDirectional	−0.01	0.09	−0.19	0.18
PerspectiveSelf: ConsistencyConsistent	0.12	0.04	0.04	0.20
PerspectiveSelf: TypesofCueSocialPDirectional	0.10	0.04	0.02	0.18
ConsistencyConsistent: TypesofCueSocialPDirectional	0.04	0.04	−0.03	0.12
PerspectiveSelf: ConsistencyConsistent:TypesofCueSocialPDirectional	−0.14	0.06	−0.25	−0.03

### Error rate analysis

**Figure 6.**
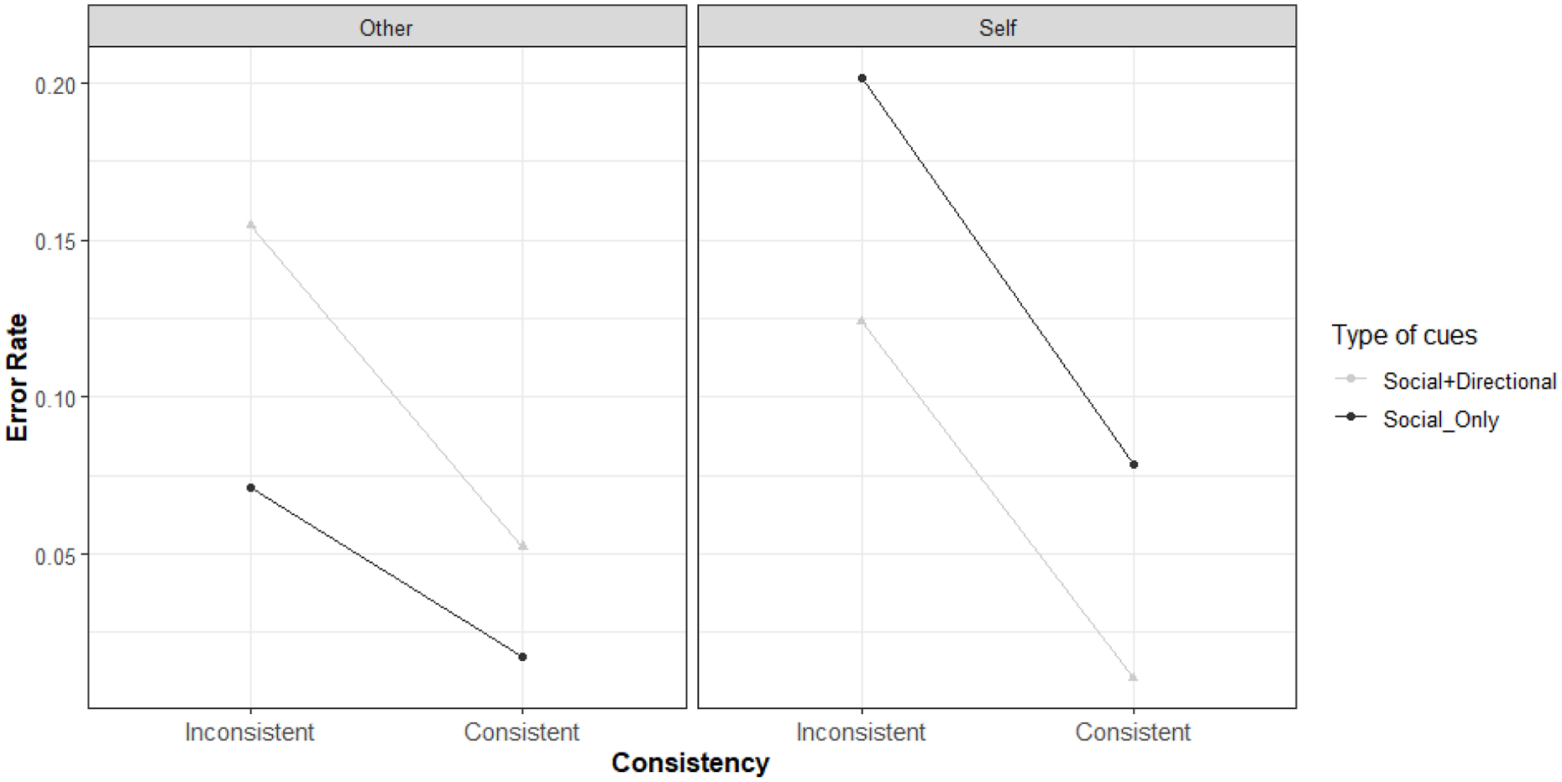
Estimated marginal means of the error rate for each combination of Consistency (inconsistent vs. consistent) and Perspective (self vs. other), split for Types of cue (Social+Directional vs. Social_Only).

Table 3 shows the results of the Bayesian mixed-effects model and Figure 6 shows the estimated marginal means of the interaction between Consistency and Perspective split by the two types of cue. A main effect of Perspective emerged, with a higher error rate for the Self condition (1.21, *SE* 0.37, 95% CI [0.49, 1.94]). A main effect of Consistency also emerged, with a lower error rate for the Consistent trial (−1.49, *SE* 0.62, 95% CI [−2.81, −0.34]). In addition, an interaction effect between Perspective and Types of cue emerged, with a lower error rate in the Self—*Social+Directional* condition (−1.47, *SE* 0.50, 95% CI [−2.45, −0.50]).

**Table 3.** Population-level effects of the brms model.

Covariate	Estimate	Est. error	l-95% CI	u-95% CI
Intercept	−2.58	0.41	−3.42	−1.80
PerspectiveSelf	1.20	0.37	0.50	1.95
ConsistencyConsistent	−1.50	0.61	−2.78	−0.37
TypeofCueSocialPDirectional	0.88	0.55	−0.19	1.97
PerspectiveSelf: ConsistencyConsistent	0.42	0.70	−0.91	1.84
PerspectiveSelf: TypesofCueSocialPDirectional	−1.46	0.51	−2.48	−0.50
ConsistencyConsistent: TypesofCueSocialPDirectional	0.30	0.74	−1.11	1.78
PerspectiveSelf: ConsistencyConsistent:TypesofCueSocialPDirectional	−1.84	1.06	−4.00	0.22

### Planned post hoc comparisons

Because predictors in models are conditional to all other factors with which they interact, they do not provide the desired comparisons. As specified in the introduction, to assess the mentalising and the domain-general accounts only the Self level of the Perspective variable is relevant. Within this level of the Perspective variable, we conducted the following comparisons:

- Inconsistent vs. consistent within the *Social+Directional* type of cue;
- Inconsistent vs consistent within the *Social_Only* type of cue;
- Between the interferences of the two cues (inconsistent–consistent in the *Social+Directional* cue vs. inconsistent–consistent in the *Social_Only* cue).

Post hoc comparisons were extracted using the *emmeans* package version 1.5.4 (Lenth, 2021) and the *Easystats* package version 0.2.0 (Lüdecke et al., 2020). Decisions on the comparisons were based on the relative positions of the highest density interval (HDI, Box & Tiao, 1992; Chen et al., 2000; Hespanhol et al., 2019) and the predefined regions of practical equivalence (ROPE) of 89% (Kruschke & Liddell, 2018a, 2018b; McElreath & Safari, 2020). In agreement with Kruschke and Liddell (2018a), the ROPEs were defined as ±.1 × *SD* for the contrasts.

### Reaction time analysis

**Figure 7.**
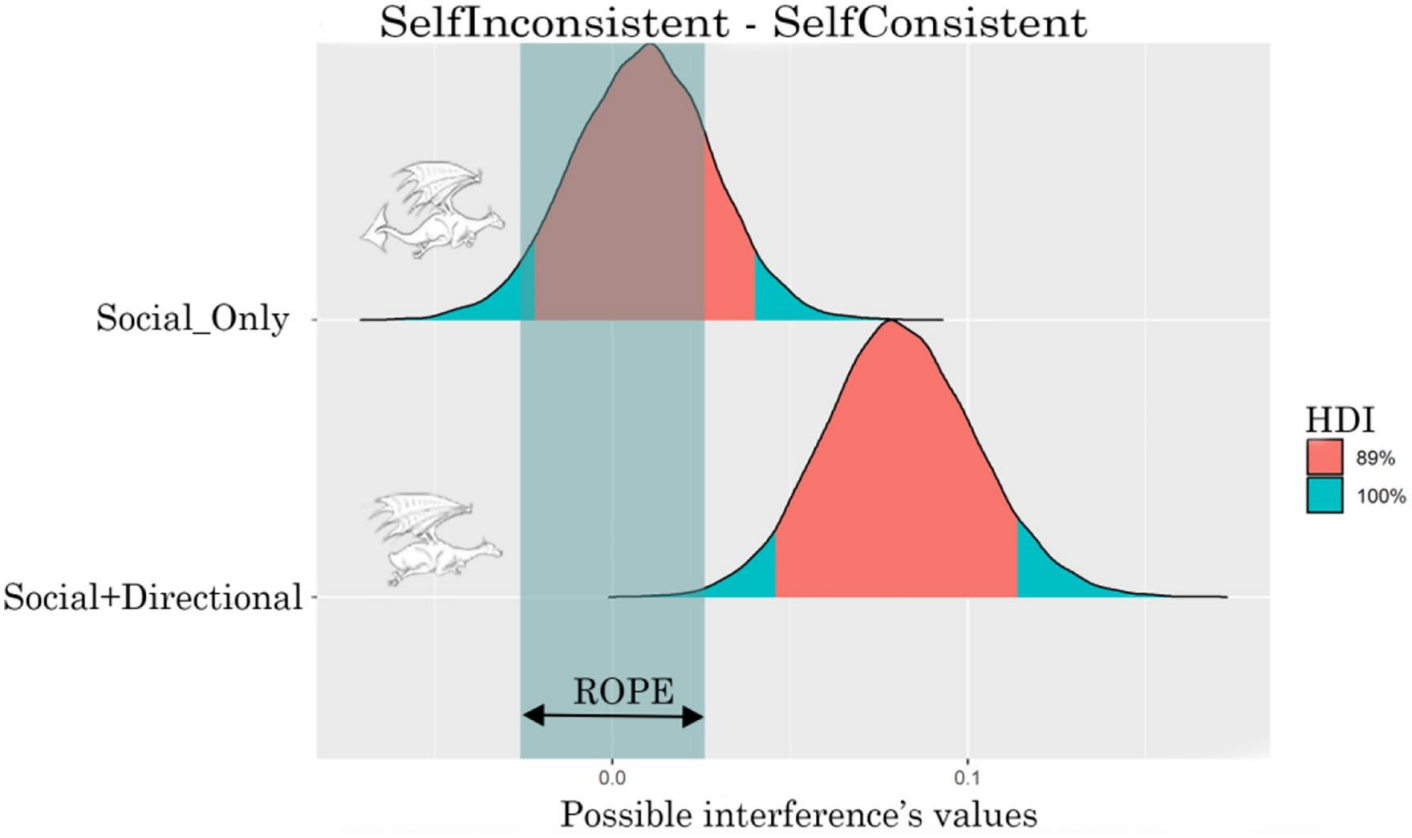
ROPE and HDI of the interaction for Social_Only and Social+Directional cues for RTs.

Table 4 and Figure 7 show the interference (intended as the RTs difference between the inconsistent and consistent levels of Consistency variable) generated by the two cues. The *Social+Directional* cue clearly generates an interference; the entire HDI falls outside of the ROPE indicating that 89% of the most credible values of the interference are different from the null value. There is instead only a 15% probability that the *Social_Only* cue generates interference; 85% of the HDI falls within the ROPE, indicating that 85% of the most credible values of the interference are practically equivalent to the null value.

**Table 4.** Interference for the two types of cue.

Parameter	Mean	89% HDI	89% ROPE	% in ROPE
Inconsistent–Consistent, *Social_Only*, Self	8.50e-03	[−0.02, 0.04]	[−0.03, 0.03]	86.07%
Inconsistent–Consistent, *Social+Directional*, Self	0.08	[0.05, 0.11]	[−0.03, 0.03]	0%

**Figure 8.**
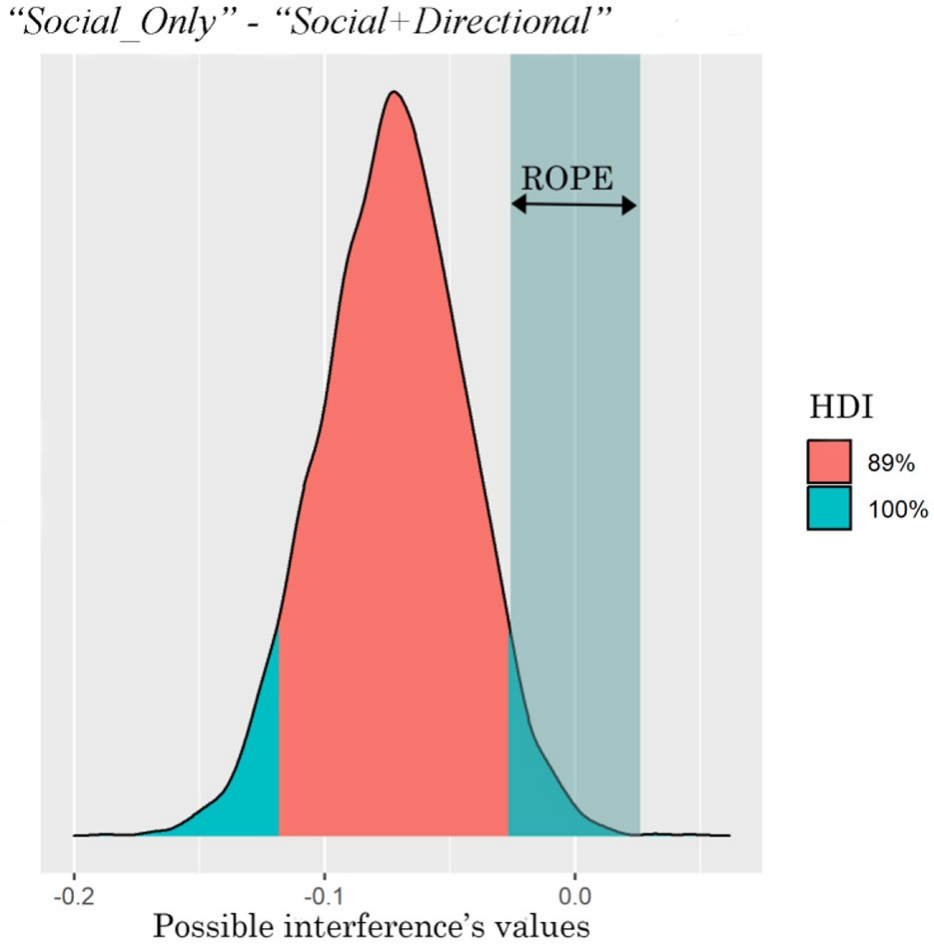
ROPE and HDI of the difference between the interferences generated by the two cues.

The comparison between the two interferences (i.e., the interferences generated by the two types of cue) is shown in Table 5 and Figure 8. It can be seen that the *Social+Directional* cue generated much more interference than the *Social_Only* cue. The entire HDI falls outside of the ROPE indicating that 89% of the most credible values of the difference between the interferences are different from the null value.

**Table 5.** Interference difference between the two cues.

Parameter	Mean	89% HDI	89% ROPE	% in ROPE
Inconsistent–Consistent, *Social_Only*—(*Social+Directional*), Self	−0.07	[−0.12, −0.03]	[−0.03, 0.03]	0%

### Error rate analysis

**Figure 9.**
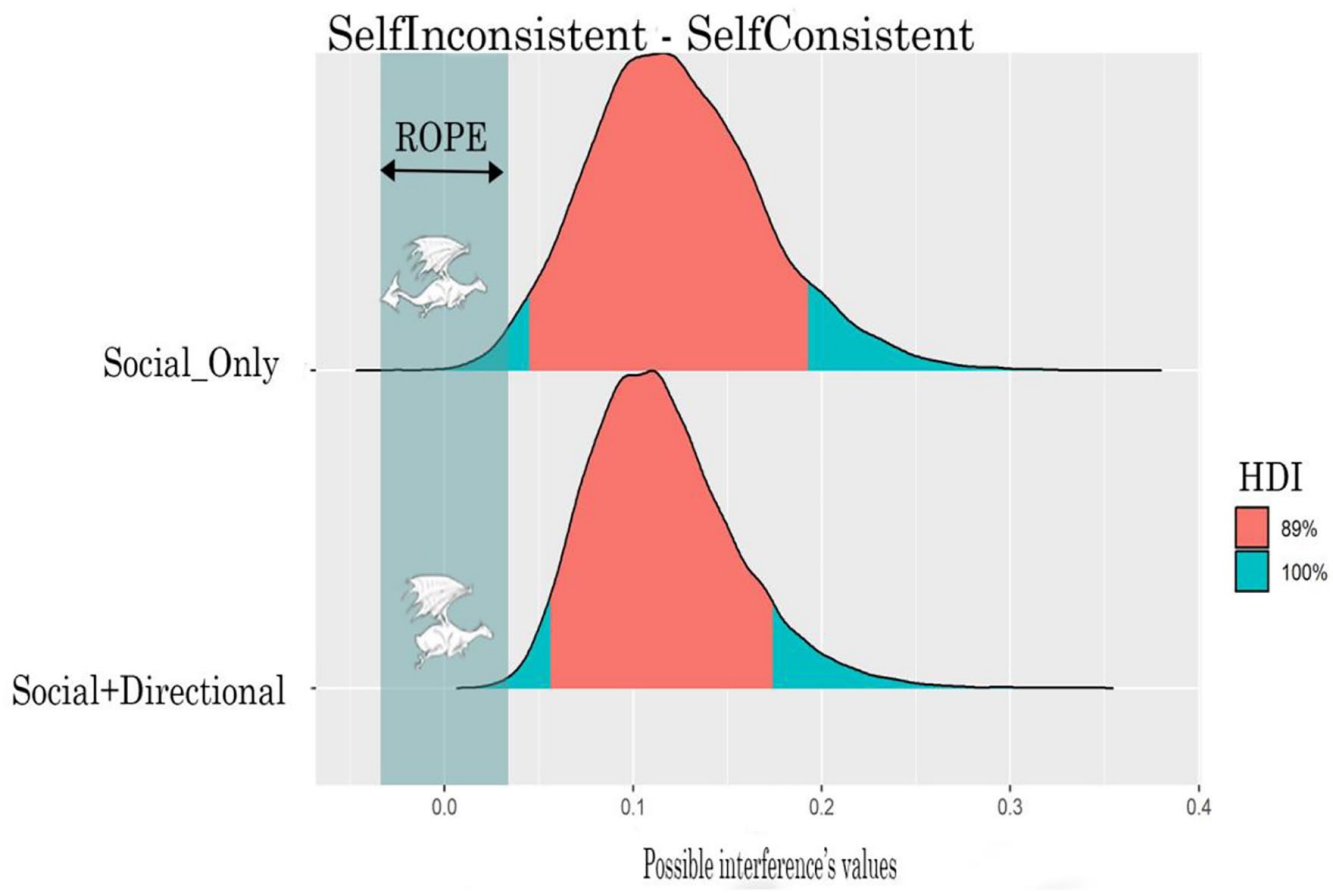
ROPE and HDI of the interaction for Social_Only and Social+Directional cues for error rate.

Table 6 and Figure 9 show the interference generated by the two cues. Both cues show an interference pattern. For both cues, the entire HDI falls outside of the ROPE indicating that 89% of the most credible values of the interference are different from the null value.

**Table 6.** Interference for the two types of cue.

Parameter	Mean	89% HDI	89% ROPE	% in ROPE
Inconsistent–Consistent, *Social_Only*, Self	0.12	[0.05, 0.20]	[−0.03, 0.03]	0%
Inconsistent–Consistent, *Social+Directional*, Self	0.12	[0.05, 0.17]	[−0.03, 0.03]	0%

**Figure 10.**
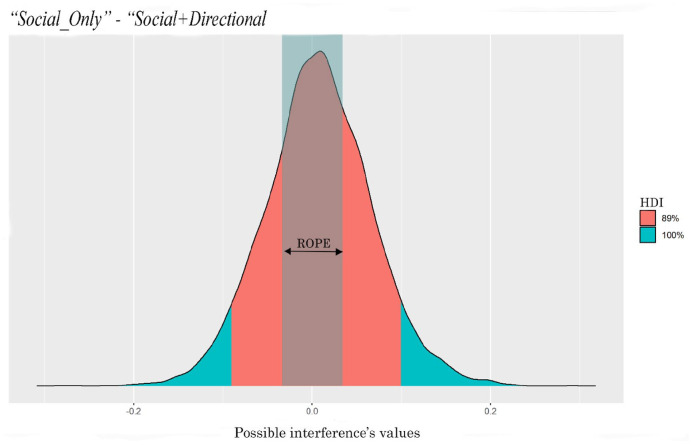
ROPE and HDI of the difference between the interference of the two types of cue.

The comparison between the interferences generated by the two types of cue is shown in Table 7 and Figure 10. It can be seen that the two types of cue generated a similar amount of interference with no evident difference between the two cues.

**Table 7.** Interference difference between the two cues.

Parameter	Mean	89% HDI	89% ROPE	% in ROPE
Inconsistent–Consistent, *Social_Only*—(*Social* *+* *Directional*), Self	7.72e-03	[−0.09, 0.11]	[−0.03, 0.03]	47.65%

### Control analysis on error rate

As the analysis of the errors was not in line with the RTs analysis, we conducted further investigations. First, we thought that the incongruence between RTs and error rate may have something to do with the arrangement of the scene. There were two types of Inconsistent trials, one in which the targets were presented all in the same wall or in two walls. The differences in Errors between the two types of trials were investigated for both cues through a Bayesian mixed-effects model. The model included the variables Consistency and Types of cue—together with their interaction—and Walls as population-level factors and the variable Subject and Trials nested within Subject as a group-level factor. Table 8 shows the results of the model. As can be seen, no effect on the number of walls emerged.

**Table 8.** Population-level effects of the brms model.

Covariate	Estimate	Est. error	l-95% CI	u-95% CI
Intercept	−0.68	0.62	−1.93	0.54
ConsistencyConsistent	−1.39	0.41	−2.21	−0.61
TypesofCueSocialPDirectional	−0.63	0.56	−1.77	0.44
Walls	−0.53	0.36	−1.23	0.16
ConsistencyConsistent: TypesofCueSocialPDirectional	−1.56	0.82	−3.28	−0.06

## Discussion

Our attention cannot help being reflexively affected by what other individuals are looking at. Two accounts have been advanced to explain this reflexive attentional shift phenomenon. The mentalising account suggests that we reflexively infer the mental state of the others and that our attention is affected by the others’ social features; that is a visual perspective-taking process (Capozzi et al., 2014; Furlanetto et al., 2016; Samson et al., 2010), while the domain-general account suggests that this phenomenon is due to the others’ directional features such as their posture and orientation (Cole et al., 2015; Langton, 2018; Santiesteban et al., 2015; Wilson et al., 2017). To compare the two accounts, we employed the dot-perspective task ideated by Samson et al. (2010). It consists of a virtual room in which targets appear and the Other is represented by a cue (usually a human avatar) pointing either consistently or inconsistently with the participant’s perspective at the location of the targets. Participants are requested to indicate as quickly as possible whether the number of targets corresponds to a prompted number. RTs and error rates are the dependent variables. Typically, this task exhibits an interference: participants are slower and do more errors in inconsistent trials.

This interference is due to participants reflexively shifting their attention towards the direction faced by the cue. Using a human avatar as a cue, however, may not be ideal to compare the two accounts because both the social and the directional features of the avatar jointly point to the same direction. Instead of a human avatar, therefore, we used a bidirectional cue represented by a dragon with an arrow-shaped tail pointing oppositely to its posture. We hypothesised that the directional features of the tail would cancel out or attenuate the directional features of the muzzle, isolating therefore the social features. This has been confirmed by a preliminary experiment (see online Supplementary Material A) which showed that the directional features of this cue have a scarce effect on orienting attention. We named this cue the *Social_Only* cue. Two clarifications must be made. The first pertains to the social features of the dragon. It may be objected that a dragon-shaped avatar is different from a human avatar as it does not resemble a human figure and it is a fantasy creature; hence, it may be claimed that it does not have any social feature. It should be noted that different studies showed that non-human animals as well as mascots and fantasy creatures do orientate attention in the same way as human avatars (Dujmović & Valerjev, 2018; MacDorman et al., 2013; Simpson & Todd, 2017). In particular, MacDorman et al. (2013) showed that the eeriness of the others does not stop people to take their perspective, whereas this can be affected by previous exposure/familiarity to them. As the dragon is present and inherited in every culture (Blust, 2000; Khalifa-Gueta, 2018), it can be assumed that it is a familiar cue in which participants can identify a viewpoint, which signifies its social feature.

Second, Nielsen et al. (2015) claimed that arrows also include some social features and should be considered as semi-social cues. This would imply that the arrow-shaped tail of the Social_Only cue should attenuate the social features in addition to its directional features. This claim is not empirically supported and contrasts with Massironi and Bruno (2001)’s explanation of the role of arrows (see Note 1). Even conceiving that arrows embed some social features, these are surely secondary to their directional features.

In our main experiment, the effects of the *Social_Only* cue were compared with those of a similar cue devoid of the tail. The directional features of this cue were not contrasted by anything else, resulting in a *Social+Directional* cue. A preliminary experiment (see online Supplementary Material A) confirmed that the directional features of this cue do direct attention.

Results of the main experiment showed a different pattern of interference between RTs and error rate. From the analysis of the RTs, it emerged that while the *Social+Directional* cue generated a strong interference, the *Social_Only* cue did not. This result clearly supports the domain-general account because the social features of the cue alone were not capable of generating the interference. It should be also stressed that the effect of the arrow-shaped tail in cancelling out the interference was particularly strong considering that the tail was irrelevant for the task. Participants in the *Social_Only* condition were never asked to pay attention to the dragon’s tail nor the tail was mentioned in the instructions or at any other moment of the experiment.

The analysis of the errors, however, was not in line with that of RTs. Inconsistency between RTs and errors is not new (see introduction). More errors were performed by participants in the inconsistent trials than in the consistent trials with both cue types. This indicates that the interference persisted even when the social features were isolated. Different from the analysis of RTs, this result supports the mentalising account. There is, however, another interesting outcome emerging from the analysis of the errors: overall, there were more errors in the *Social_Only* cue than in the *Social+Directional* cue. In the following sections, we offer an interpretation of (1) why the *Social_Only* cue generated more errors than the *Social+Directional* cue and (2) why the interference was observed for the errors but not for the RTs in the *Social_Only* cue.

### Higher number of errors in the *Social_Only* cue: speed/accuracy trade-off

In the first instance, we controlled that the higher number of errors in the *Social_Only* cue was caused by a confounding variable. In some of the inconsistent trials, the targets appear all in one wall but in others they appear in two walls. A dedicated analysis, however, showed that this was not the case: the difference of errors between the two conditions, one wall versus two walls, was similar.

The speed/accuracy trade-off, however, can explain the result. When the time constraint is short (2 s in our case) and the task is more complex, participants may focus on speed rather than accuracy. In our experiment, the *Social_Only* cue—having two contrasting directional features—can be thought of as more complex than the *Social+Directional* cue. As the speed resulted to be similar for the two cues, a decrease in accuracy must emerge in the more complex cue.

### *Social_Only* cue: Interference in errors but not in RTs

The speed/accuracy trade-off hypothesis cannot, however, explain the interference in the errors of the *Social_Only* cue. This would have generated a similar number of errors in both consistent and inconsistent trials. The presence of an interference in errors but not in RTs favours the hypothesis that the two measures reflect different processes (Kahana & Loftus, 1999; Prinzmetal et al., 2005). As mentioned in the introduction, Prinzmetal et al. suggested that attention is driven by both a voluntary process, which affects both RTs and accuracy and an involuntary process which affects RTs only. Accordingly, we suggest that the dot perspective task requires both, an (involuntary) orienting and a (voluntary) decisional process: Participants are first involuntarily oriented towards the location indicated by the directional features of the cue. Then, a voluntary decisional process confirms whether the number of targets visible from the given perspective corresponds to the prompted one. This decisional process is affected by the social features of the cue. When the social features are isolated, the elicited mentalising processes on their own have scarce or no power to direct attention; they can only affect the decisional process. This might explain why mentalising processes have not been detected by some studies (e.g., Cole et al., 2015; Conway et al., 2017; Santiesteban et al., 2015; Wilson et al., 2017; and others). Moreover, it can also explain why some other studies did not detect the interference in the error rate (Cole et al., 2016; Langton, 2018; O’Grady et al., 2020). In these cases, it can be assumed that the involuntary process driven by the directional features of the cue might have overpowered the voluntary process driven by the social features.

To sum up, when another is present in the visual scene and we are requested to validate/confirm our point of view, our attention is oriented by the other’s directional features while their social features affect our decisional processes. RTs and error rates are often employed to measure the same cognitive processes, even in studies employing the dot perspective task. Previous ambiguous results together with our findings show that this should not be always assumed.

The suggested integrated approach between the mentalising and domain-general accounts is further supported by the results originating from tasks eliciting either the decisional or the orienting process separately. For example, in Posner’s spatial cueing task (Posner & Cohen, 1984), which does not require any decisional process, Hayward and Ristic (2018) showed that a directional cue directs attention regardless of its social features. Conversely, in a task that engages only a decisional process, as in the Room Observer and Mirror Perspective test (ROMP, Bertamini & Soranzo, 2018; Soranzo et al., 2021) in which participants were asked to judge how many targets are visible from a given position indicated by a cue, an advantage emerges for social cues compared to non-social cues.

## Conclusion

To summarise, in this study, we investigated the role of social and directional features of the other in reflexively orientating attention. We developed a cue having only social features (*Social_Only* cue) and compared its effects with a cue with conjugated social and directional features (*Social+Directional* cue). Our results showed that while the *Social+Directional* cue was able to generate interference in both RTs and error rates, the *Social_Only* cue does not generate interference in the RTs but only in the error rate. We suggest that in the dot perspective task two processes are involved: an involuntary orientating process—measured by the RTs—and a voluntary decisional process—measured also by the error rate. We propose therefore an integrated approach between the mentalising and the domain-general accounts to explain the reflexive attentional shift emerging in the dot-perspective task.

## Supplemental Material

sj-docx-1-qjp-10.1177_17470218221094310 – Supplemental material for Both the domain-general and the mentalising processes affect visual perspective taking Click here for additional data file. Supplemental material, sj-docx-1-qjp-10.1177_17470218221094310 for Both the domain-general and the mentalising processes affect visual perspective taking by Gabriele Pesimena and Alessandro Soranzo in Quarterly Journal of Experimental Psychology

## Notes

1 Nielsen et al. (2015) suggested that the arrows should be considered as “semi-social” cues. In support of their claim, the authors refer to the works of Kingstone et al. (2004), Ristic et al. (2002), and Zwickel (2009). However, it is unclear how these works support this claim. These works show that both arrows and social cues direct attention. In addition, and most importantly, Nielsen et al.’s claim contrasts with Massironi and Bruno (2001)’s explanation: “The communicative power of arrows lies in the fact that they can convey information about orientation, intensity, and direction of a force, and they can do so in non-ambiguous, perceptually eloquent fashion, these perceptual features are readily detected by low-level, bottom-up visual processes” (p. 167).

2 The effects of the Social_Only cue were not compared, instead, with those of a dragon without the muzzle (e.g., a “*Directional_Only*” cue). This condition would have been superfluous for the aim of comparing the two accounts. As pointed out by Cole and Millet (2019), showing that a directional-only cue generates interference does not rule out the mentalising account because different processes may give rise to a similar effect.

3 The scenario in which the interference generated by the *Social+Directional* cue is smaller than that generated by the *Social_Only* cue is not plausible. It would mean that the arrow-shaped tail orients attention opposite to where it points.

4 Note that Wilson et al. (2017) found that impersonal pronouns generate the same amount of interference as personal pronouns therefore the prompt DRAGON was used (see also MacDorman et al., 2013).
