# Supplementary material for: Both the domain-general and the mentalising processes affect visual perspective taking
Source: Q J Exp Psychol (Hove). 2022 Jun 2;76(3):469–84. doi: 10.1177/17470218221094310 (PMC9936435; doi:10.1177/17470218221094310)
Supplement: sj-docx-1-qjp-10.1177_17470218221094310 – Supplemental material for Both the domain-general and the mentalising processes affect visual perspective taking [file sj-docx-1-qjp-10.1177_17470218221094310.docx]

Supplementary Material A for:

**Both the domain-general and the mentalizing processes affect visual perspective taking.**

Gabriele Pesimena & Alessandro Soranzo

**Introduction and Methods**

Two preliminary experiments were conducted to assess whether the *Social_Only* and *Social+Directional* cues orient attention. For this purpose an adapted version of the Posner paradigm (Posner & Cohen, 1984) was used. The experimental sequence of the events was as follow: At the beginning, a fixation cross was presented at the centre of a computer screen for 1000 ms. This was then followed by the onset of a cue (*Social_Only* cue in experiment 1A and *Social+Directional* cue in experiment 1B) in the centre of the screen for 200 ms. A target disc was then presented either to the left or to the right. Participants were asked to press the “LEFT” key on their keyboard when the target appeared on the left and the “RIGHT” key when the target appeared on the right. A total of 128 trials were presented in a random order. As in a typical Posner paradigm, in 75% of the trials the cue faced towards the location of the target (Congruent) and to the opposite direction in the remaining 25% (Incongruent). Reaction times were recorded.

After 64 trials participants were requested to have a break in order to avoid any fatigue effect. Before starting the experiment participants underwent a short practice.

32 Participants in total (16 per experiment, see methods section in the main study for the power analysis) took part in this study (age range 22 to 55 years old) of which 16 females. Participants were naïve to the purpose of the study and received no remuneration for taking part. None of these participants took part in the main study. See main study for recruitment and ethics procedure.

**Results**

For a description of the statistical tools employed in this preliminary study see the main study results section. Means and standard deviations for RTs are shown in Table A1 and Figure A1

Table A1

*Mean and sd for each condition for each Types of Cue*

| Congruency | Types of Cue | Mean (seconds) | sd |
| --- | --- | --- | --- |
| Incongruent | *Social_Only* | 0.492 | 0.131 |
|  | *Social+Directional* | 0.535 | 0.225 |
| Congruent | *Social_Only* | 0.477 | 0.143 |
|  | *Social+Directional* | 0.474 | 0.215 |

As it can be seen in table A1, participants were faster in the Congruent than in the Incongruent condition for both *Social_Only* and *Social+Directional* cue. However, a meaningful difference is evident between the two cues. Participants were overall slower in the Incongruent condition of the *Social+Directional* cue.

Table A2 shows the results of a Bayesian Weibull mixed-effects model with Congruency and Types of cue - together with their interaction - as population-level factors and Subject as group-level factor. Flat priors for the population level effects were used and weakly informative priors for the intercept [student_t(3, 0.7, 2.5)] and for the group level effects [student_t(3, 0, 2.5)]. For model estimation, four chains with 5000 iterations (2500 warmup) were used. Convergence was checked as for the main study.

Table A2

*Population level effects of the brms model*

| Covariate | Estimate | Est.Error | l-95% CI | u-95% CI |
| --- | --- | --- | --- | --- |
| Intercept | -0.76 | 0.07 | -0.89 | -0.63 |
| CongruencyCongruent | -0.02 | 0.02 | -0.05 | 0.01 |
| TypesofCue*Social+Directional* | 0.12 | 0.09 | -0.06 | 0.29 |
| CongruencyCongruent:TypesofCue*Social+Directional* | -0.08 | 0.02 | -0.13 | -0.04 |


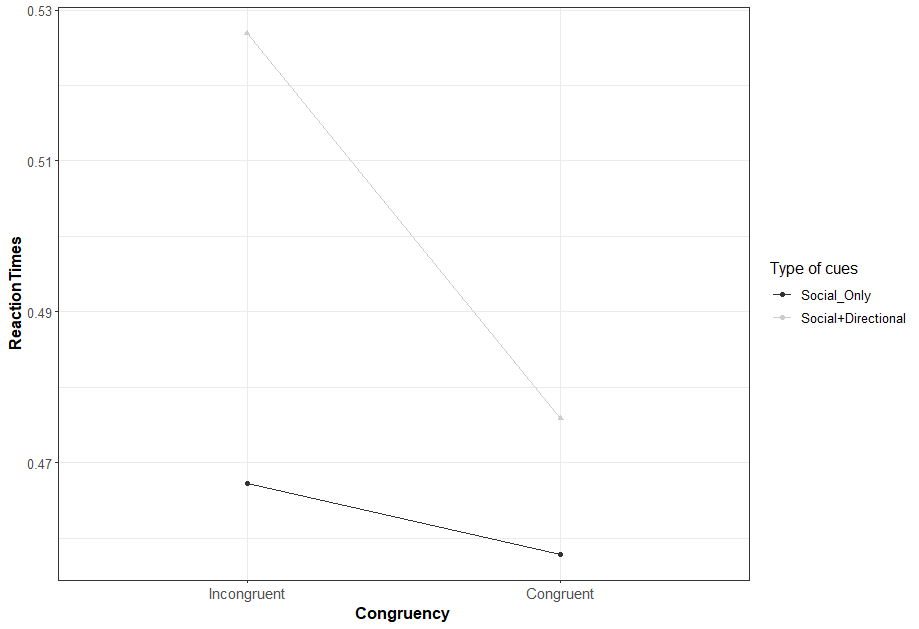


*Figure A1.* Estimated marginal means for each level of Congruency (Inconsistent vs Consistent) for each Types of cue (Social+Directional vs Social_Only).

Figure [A1](#_heading=h.1y810tw) and table A2 show the estimated marginal means of the two different conditions (Congruent vs Incongruent) for the two cues (*Social+Directional* and *Social_Only*). A main effect of the interaction between Types of Cue and Congruency emerged, with shorter RTs for the Congruent and *Social+Directional* trials [ -0.08, SE 0.02, 95% CI (-0.13, -0.04)].

**Cueing effects of the two cues**

Table A3 and Figure A2 show the cueing effects (intended as the mean difference between the Incongruent and Congruent conditions) generated by the two types of cue. As it can be seen, the *Social+Directional* cue clearly generates a cueing effect; with the entire HDI falling outside the ROPE. While the *Social_Only* cue does not with 92.8% of the HDI falling within the ROPE.

Table A3

*Cueing effect for each types of cue.*

| Parameter | Mean | 89% HDI | 89% ROPE | % in ROPE |
| --- | --- | --- | --- | --- |
| Incongruent - Congruent, *Social_Only* | 0.001 | [0.00, 0.02] | [-0.02, 0.02] | 92.79% |
| Incongruent – Congruent, *Social+Directional* | 0.05 | [ 0.04, 0.07] | [-0.02, 0.02] | 0% |


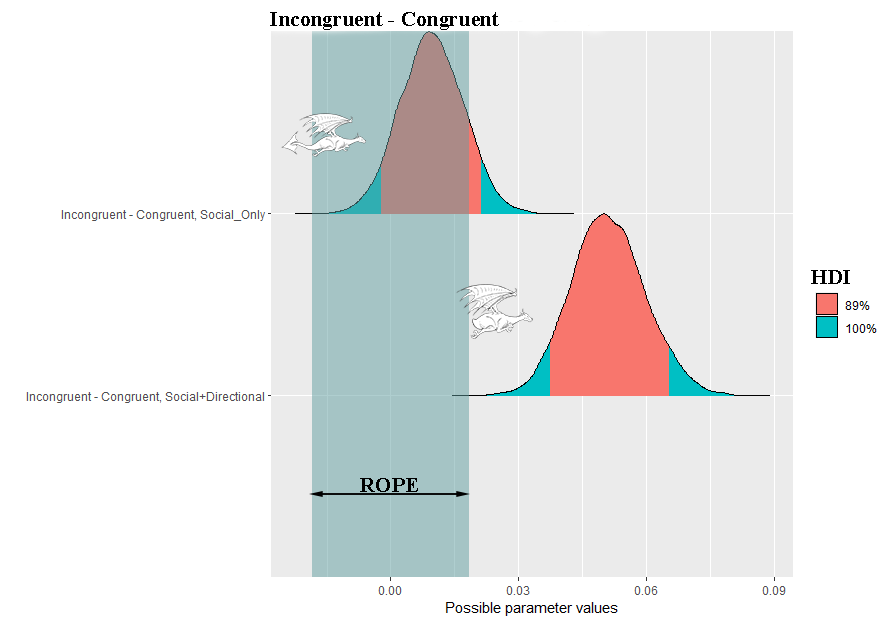


*Figure A2.* ROPE and HDI of the interaction for the two types of cue: Social_Only and Social+Directional cue.

Taken together these results show that the *Social+Directional* cue orients attention while the *Social_Only* cue does not. This indicates that the arrowed-shaped tail cancels out (or at least attenuates) the directional features of the dragon’s posture.
